# Urinary miR-21 as a potential biomarker of hypertensive kidney injury and fibrosis

**DOI:** 10.1038/s41598-017-18175-3

**Published:** 2017-12-18

**Authors:** Congcong Chen, Chaosheng Lu, Yan Qian, Haiyan Li, Yi Tan, Lu Cai, Huachun Weng

**Affiliations:** 1 Chinese-American Research Institute for Pediatrics & Department of Pediatrics, The First Affiliated Hospital of Wenzhou Medical University, Wenzhou, Zhejiang China; 2 Department of Pharmaceutical Sciences, Wenzhou Medical University, Chashan University-town, Wenzhou, Zhejiang China; 3 Pediatric Research Institute, Departments of Pediatrics, Radiation Oncology, Pharmacology and Toxicology University of Louisville, Louisville, Kentucky USA

## Abstract

Kidney biopsy is considered the golden criterion for diagnosing the etiology of kidney disease but accompanied by non-negligible complications. We explored the possibility of using urinary microRNA (miRNA) as a non-invasive biomarker for hypertensive kidney injury. We assessed differential miRNA expressions in the kidneys and urine of hypertensive mice with kidney injury induced by deoxycorticosterone acetate (DOCA)-salt compared to the controls. DOCA-salt treatment significantly increased renal tubular lesions from day 2 and mRNA expression of fibrosis-related genes from day 4 compared to the controls, respectively. Urinary albumin and N-acetyl-beta-D-glucosaminidase was significantly increased on day 8 compared to the controls. Array results showed that 20 out of 585 miRNAs were highly expressed in the kidneys and significantly increased on day 8 compared to the controls, including miR-21, miR-146b, miR-155 and miR-132, which were confirmed by real-time polymerase chain reaction and were significantly higher from day 4. The miR-21/creatinine in the urine from day 4 was significantly higher than that of the controls and was detected earlier than urinary albumin. In conclusion, we have identified urinary miR-21 that correlates with histopathological lesions and functional markers of kidney damage to facilitate a potential noninvasive detection for hypertensive kidney injury.

## Introduction

Hypertension is a major risk factor for stroke, myocardial infarction, and kidney failure^1^. To date, clinical biomarkers of renal function and injury primarily relies on several limited tools, such as proteinuria and urinary sediment analysis, to help anticipate various etiologies of underlying renal disease, including hypertensive kidney disease, but may not pin-point a specific diagnosis or degree of activity^2,3^. Kidney biopsy has been considered a golden criterion and provides valuable information regarding diagnosis, prognosis, and treatment decisions^4^. However, kidney biopsies are an invasive procedure with non-negligible complications^5^. Noninvasive markers of kidney disease etiology could promote clinical medicine by replacing a currently invasive procedure and improve diagnostic accuracy in patients who do not undergo a biopsy.

MicroRNAs (miRNAs) are a family of short, non-coding RNAs that are approximately 22–25 nucleotides long and that bind to complementary sequences in the 3′ untranslated regions of their target mRNAs and induce mRNA degradation or translational repression^6,7^. Recent findings revealed that miRNAs play important roles in various physiological and pathologic processes^8–11^. Furthermore, expression changes of miRNAs have been reported in renal disease, including renal tumors^12^, diabetic nephropathy^13,14^, immunoglobulin-A nephropathy^15^, and acute rejection after renal transplantation^16,17^. Circulating miRNAs have been shown to be useful as diagnostic biomarkers not only in kidney disease but also in various cancer, liver disease, and myocardial injuries^10,18,19^. Some qualities, such as abundant expression, tissue specificity, stability, and evolutionary conservation make extracellular miRNAs attractive as noninvasive biomarkers that reflect disease states^20^.

miRNA originating from specific tissues can be secreted into the extracellular environment, such as biological fluids^21^. Urine is one of the biological fluids comprising end-products generated by the kidneys and can be collected noninvasively and in a simple manner. The majority of urinary miRNA originates from renal cells, and analysis of these cells can provide a measure of the health of the excretory system^22–24^. In this study, we assessed differential expression levels of miRNAs in the kidneys and urine of deoxycorticosterone acetate (DOCA)-salt induced hypertensive mice compared to the controls to determine the worthy miRNAs in the early detection of hypertensive kidney injury and fibrosis.

## Results

### General features of DOCA-salt induced renal damage

**Figure 1:**
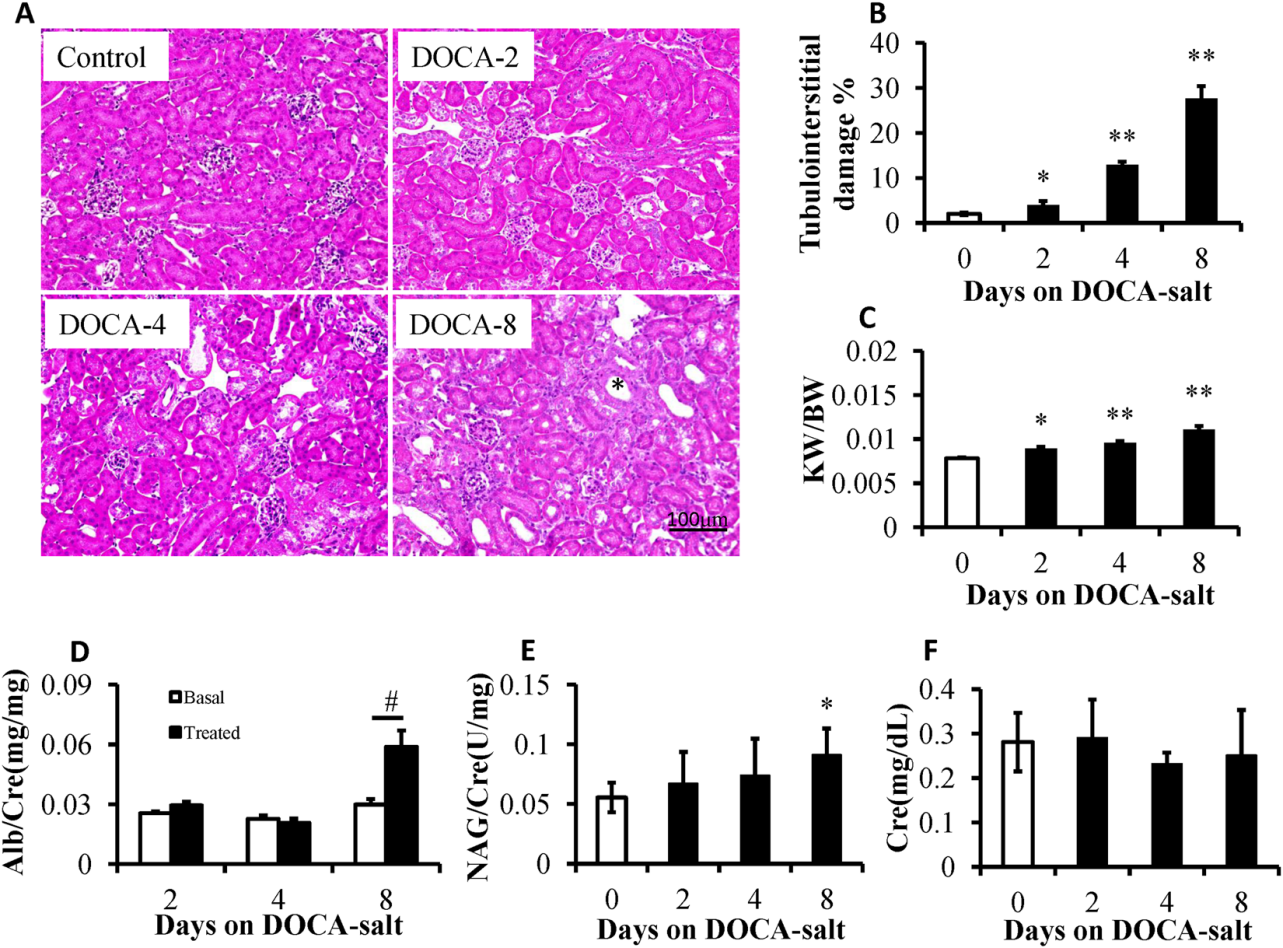
Deoxycorticosterone acetate (DOCA)-salt treatment induced tubulointerstitial lesions. (**A**) Representative images of hematoxylin-eosin-stained kidney sections of mice under control conditions and DOCA-salt-treated conditions for 2 days (DOCA-2), 4 days (DOCA-4), and 8 days (DOCA-8). DOCA-treated mice showed gradually aggravated tubular dilatation/atrophy (*), epithelial cell necrosis, and interstitial edema. The area of tubulointerstitial injury, as well as the entire cortical area, in 10 high-power fields was measured with ImageJ software. (**B**) The degree of tubulointerstitial injury was evaluated as a ratio relative to the entire cortical area. (**C**) The ratio of kidney weight (KW)/body weight (BW) was increased after DOCA-salt treatment. (**D**,**E**) Urinary albumin (Alb) and N-acetyl-beta-D- glucosaminidase (NAG) levels were elevated by the treatment with DOCA-salt. Serum creatinine (Cre, **F**) levels in the mice treated with DOCA-salt for 8 days were comparable to the control mice. *P < 0.05, **P < 0.01 compared to the basal condition; ^#^ *P* < 0.01; n = 8, error bars, mean ± SD.

DOCA-salt treatment increases blood pressure and results in renal damage, especially tubular lesions in the kidney. We assessed the morphology in the kidneys of mice treated with DOCA-salt for 0, 2, 4, 8 days. As shown in Fig. 1A–C, an increase of renal tubular lesions and renal weight/body weight was initially observed from day 2 of DOCA-salt treatment and was gradually exacerbated on day 4 or day 8. Urinary albumin excretion was usually used as an indicator of renal damage. As demonstrated in Fig. 1D, urinary albumin excretion was significantly increased on day 8 of the DOCA-salt treatment compared to the controls but not on day 2 or day 4. Furthermore, urinary N-acetyl-beta-D-glucosaminidase (NAG) was significantly increased on day 8 of the DOCA-salt treatment compared to the controls (Fig. 1E). However, serum creatinine levels in the mice treated by DOCA-salt for 8 days appear not significantly changed compared to the controls (Fig. 1F).

**Figure 2:**
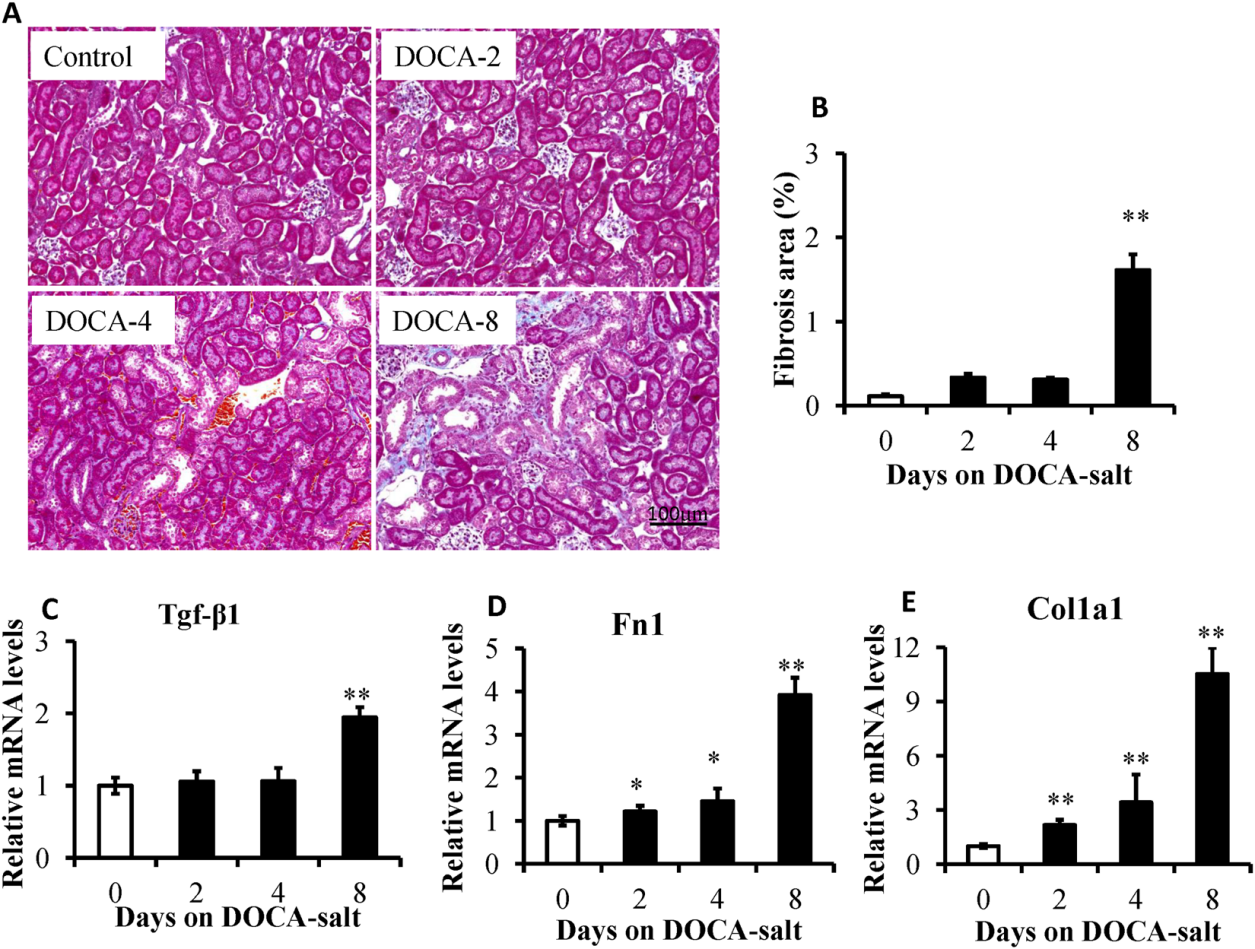
Deoxycorticosterone acetate (DOCA)-salt treatment induced tubulointerstitial fibrosis. (**A**) Representative images of Masson trichrome-stained kidney sections of mice under control conditions and DOCA-salt-treated conditions for 2 days (DOCA-2), 4 days (DOCA-4), and 8 days (DOCA-8). DOCA-treated mice showed gradually aggravated interstitial fibrosis (blue). The area of tubulointerstitial fibrosis, as well as the entire cortical area, in 10 high-power fields was measured with ImageJ software. (**B**) The degree of tubulointerstitial fibrosis was evaluated as a ratio relative to the entire cortical area, n = 8. (**C**–**E**) RT-PCR analysis of the fibrosis-related gene (transforming growth factor β1, Tgf-β1; collagen type I alpha1, Col1a1; fibronectin 1, Fn1) expression in the kidneys of the controls and mice treated with DOCA-salt for 2, 4, and 8 days, n = 5–6. **P < 0.001 compared to the controls, 5 s rRNA is used as an internal control, error bars, mean ± SD.

**Figure 3:**
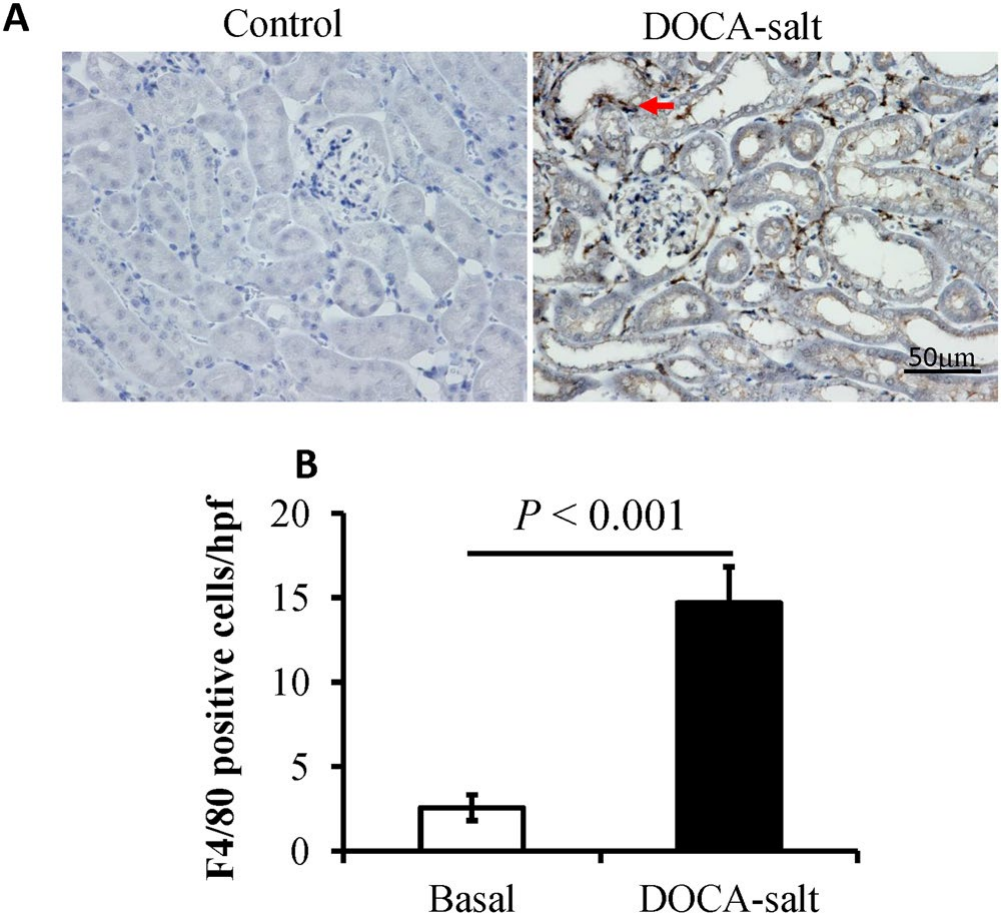
Deoxycorticosterone acetate (DOCA)-salt treatment aggravates macrophage infiltration. (**A**) Representative images of F4/80 immunohistochemical analysis in the renal cortex of the mice treated by DOCA-salt for 8 days and control mice. Macrophage (arrows) infiltration was more prominent in the tubulointerstitial region in the cortex of the mice treated by DOCA-salt for 8 days. (**B**) The number of F4/80-positive cells in 10 high-power fields (hpf) was counted. **P < 0.001 compared to the controls, n = 8, error bars, mean ± SD.

Renal fibrosis is the common feature of renal disease and is generally characterized either by an interstitial extracellular matrix or myofibroblast accumulation^25^. Masson’s trichrome staining was performed. As shown in Fig. 2A, the cortical area of mice treated by DOCA-salt for 8 days showed more severe tubulointerstitial fibrosis compared to the controls. There were significantly higher ratios of fibrous area/visual field on day 8 of the DOCA-salt treatment compared to the controls but not on day 2 or day 4 (Fig. 2B). Transforming growth factor-beta1 (Tgf-β1) is a well-known master cytokine/growth factor in fibrosis^26,27^. Tgf-β1 mRNA expression levels were significantly increased on day 8 of DOCA-salt treatment compared to the controls but not on day 2 (Fig. 2B). However, collagen type I alpha1 and fibronectin 1 mRNA expression levels were significantly increased from day 2 of the DOCA-salt treatment compared to the controls. As shown in Fig. 3, there was a greater extent of macrophage infiltration on day 8 of the DOCA treatment compared to the controls.

### Changes of miRNAs in the renal lesions and fibrosis induced by DOCA-salt

**Figure 4:**
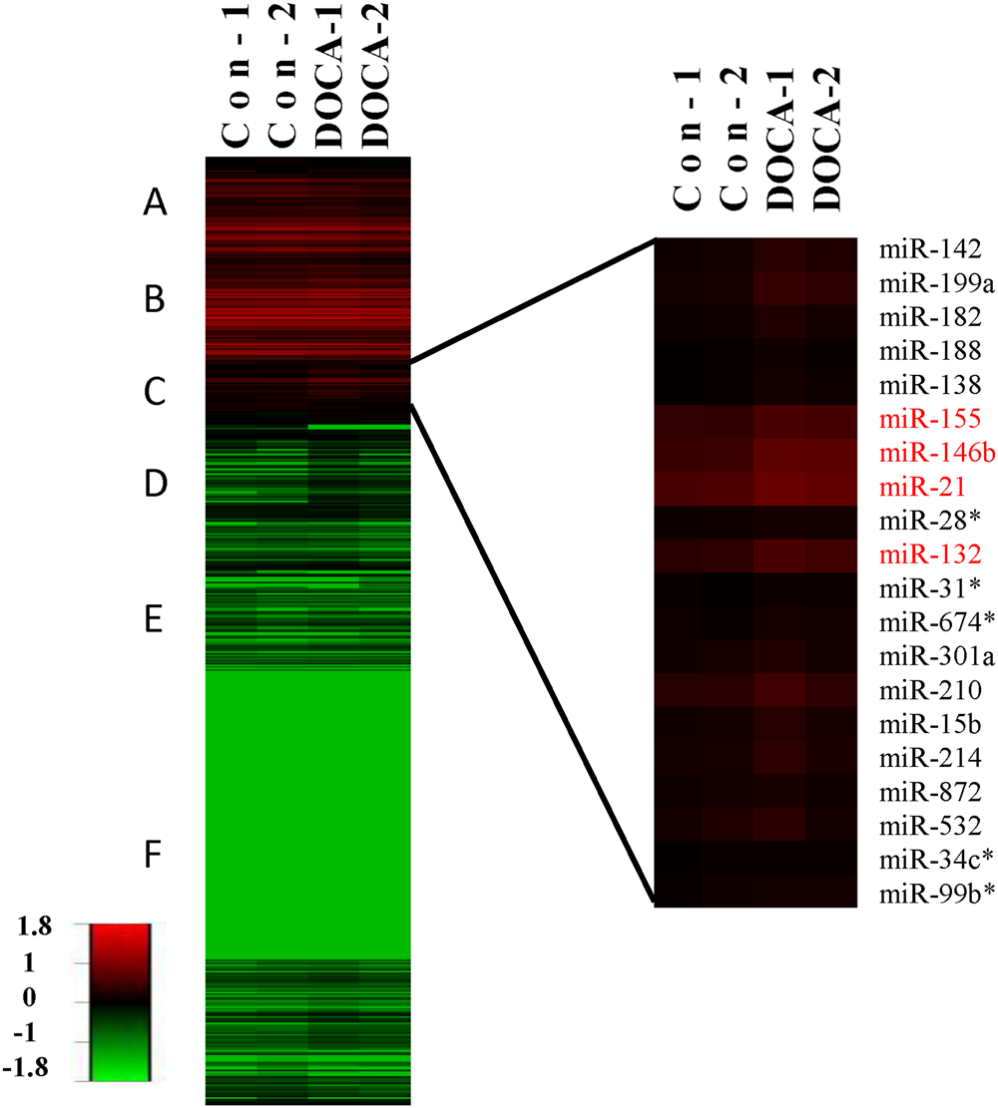
Heat map of miRNA expression in mouse kidneys. The expression profiles of 585 miRNAs in the kidneys of mice treated with deoxycorticosterone acetate (DOCA)-salt and the controls were examined with miRNA array analysis. The average linkage and Euclidean distance as the similarity measure was used. Higher expression levels are colored red, while lower levels are colored green.

To test whether renal lesion and fibrosis induced by DOCA-salt is associated with a distinctive miRNA signature, renal samples from mice treated by DOCA-salt for 8 days and the controls were screened for a total of 588 mature rodent and mouse miRNAs. A total of 3 miRNAs were not detected among the 588 mature rodent and mouse miRNAs analyzed in the study subjects (2 control and 2 DOCA-salt mice). Therefore, 585 miRNAs were actually analyzed in this study. Hierarchical clustering of the miRNAs detected on day 8 of DOCA-salt treatment and the controls showed 6 distinct clusters, A to F (Fig. 4). Cluster A, B and C contained highly expressed miRNAs in both the control and the DOCA-salt treatment. The lower expressions of miRNAs were found in clusters D, E, and F. The expression levels on day 8 of the DOCA-salt were higher than those in the controls as shown in clusters C and D. In contrast, clusters A and E included genes that were downregulated in the DOCA-salt treatment. The expression of 20 miRNAs out of 585 miRNAs in cluster C was found to be significantly increased after the DOCA-salt treatment, including miR-21, -146b, -155 and -132.

**Figure 5:**
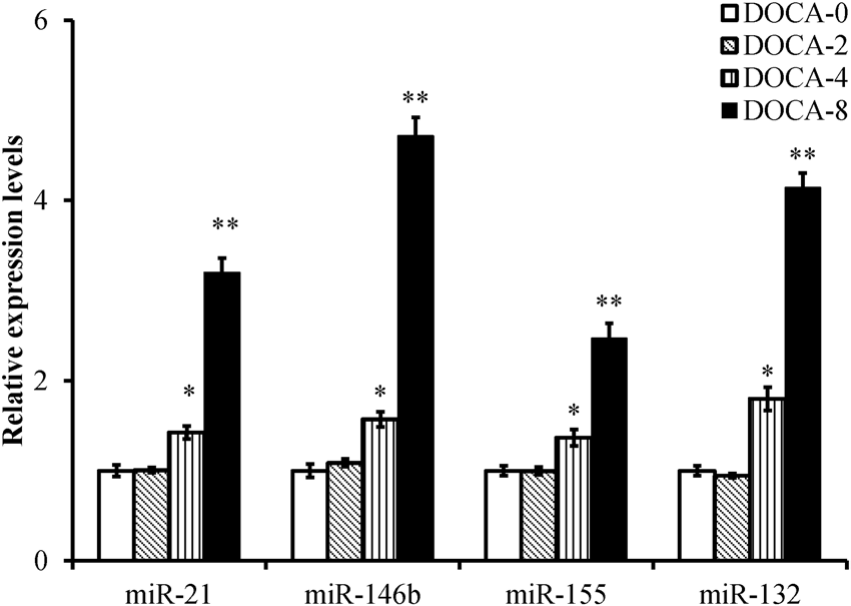
Validation of different miRNA expression in mouse kidneys. miR-21, -146b, -155, and -132 expression levels were measured in the kidneys of mice treated with deoxycorticosterone acetate (DOCA)-salt for 0, 2, 4, and 8 days. *P < 0.05, **P < 0.001 compared to DOCA-0, n = 8, 5 s rRNA is used as an internal control, error bars represent mean ± SD.

To validate the microarray data, miR-21, -146b, -155 and -132 were selected from the miRNAs that were differentially expressed in the microarray analysis and were further analyzed in kidneys by quantitative RT–PCR. As shown in Fig. 5, the expression levels of miRNA-21, -146b, -155 and -132 were significantly higher from day 4 of the DOCA-salt treatment than those in the controls, which is consistent with those in the microarray analysis.

### Association of urinary miR-21 with renal injures

**Figure 6:**
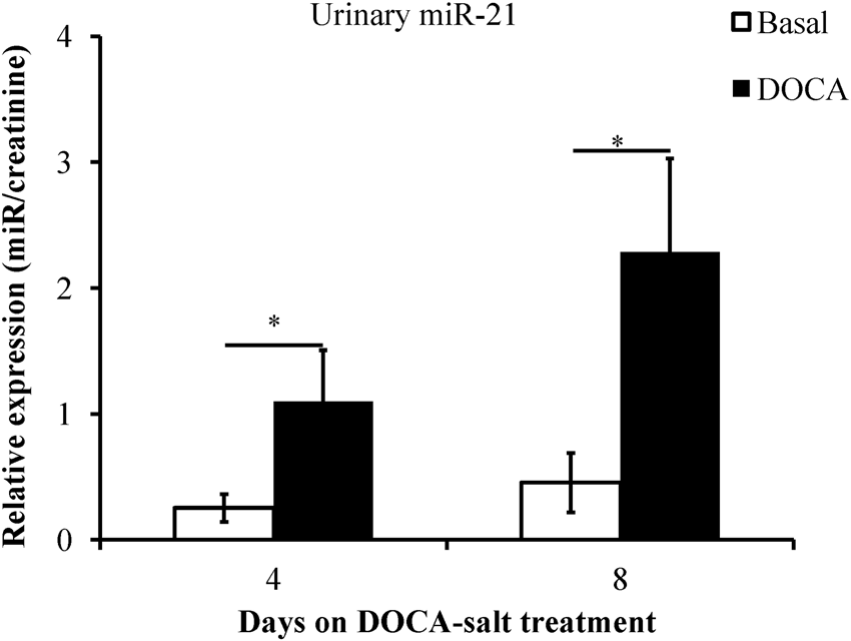
miR-21 relative expression in urine. Urinary miR-21 levels were measured in mice treated with deoxycorticosterone acetate (DOCA)-salt for 4 and 8 days. The ratios of the miR-21 and creatinine concentration were used as an evaluation index. *P < 0.05 compared to the basal conditions, n = 8, 5 s rRNA is used as an internal control for quantifying miRNAs, error bars represent mean ± SD.

Considering that previous has mentioned the increase of miR-21 in the blood of patients with renal dysfunction^28^, we furthermore, assessed the miR-21 expression levels of the 24-hour urine sample. To adjust for urine dilution, relative levels of miRNA expression were divided by urinary creatinine. The ratios of miR-21/creatinine in the urine on day 4 or day 8 were significantly higher than those of the controls (*P* < 0.05; Fig. 6).

**Figure 7:**
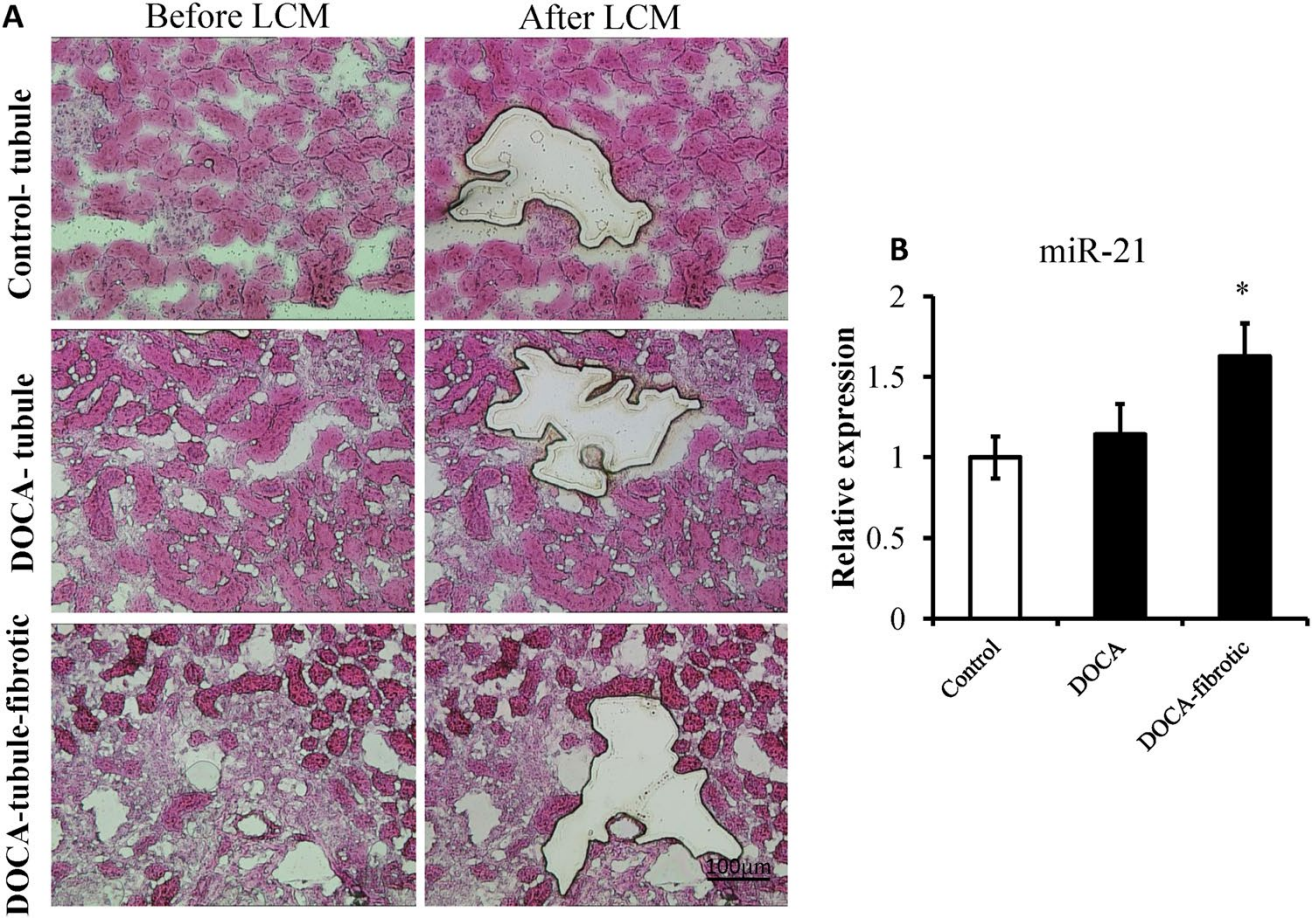
Differential expression miR-21 in fibrotic and non-fibrotic tubules. The region of fibrotic and non-fibrotic tubules in the kidneys of mice treated with deoxycorticosterone acetate (DOCA)-salt for 8 days or the controls was collected using laser capture microdissection (LCM). (**A**) Representative images of kidney sections before and after LCM. (**B**) The levels of mRNA-21 expression in fibrotic and non-fibrotic tubules in the mice treated with DOCA-salt and tubules in the controls were measured, and 18 s rRNA is used as internal control. *P < 0.05 compared to the controls, n = 3, error bars represent mean ± SD.

To verify the miR-21 generated from the region of the tubulointerstitial injury and fibrosis, Laser capture microdissection (LCM) was used to collect the regions from the renal tubule of control mice and tubulointerstitial injury and fibrosis of DOCA-salt treated mice. The miR-21 levels from the region of tubulointerstitial injury and fibrosis were significantly higher than those from the tubular control (*P* < 0.05; Fig. 7).

## Discussion

The advantages of using urine as a diagnostic tool include its noninvasive collection and its relative quickness and cost-efficiency compared to the collection of other clinical samples, such as blood. To date, monitoring or diagnosis of the process of hypertensive kidney injury relies primarily on proteinuria in combination with serum or urine creatinine. However, these biomarkers substantially change only in established kidney injuries and do not allow very early detection that is critical for effective prevention and therapy of kidney injuries. Our data show that DOCA-salt-induced hypertension and renal damage, which is characterized with albuminuria, tubulointerstitial injury, inflammatory cell infiltration and fibrosis is concerned with specific miRNA expression. Several miRNAs, especially miR-21, in urine and tissue, were found to be significantly increased after the DOCA-salt treatment for 4 days, and detected earlier than proteinuria. These results identify new urine miRNAs as sensitive and specific noninvasive biomarkers carrying a histopathologic signature of hypertensive kidney disease and may obviate the need for invasive tests.

Urinary miRNAs are approximated for the injury due to their generation from the glomeruli and tubule, which present tissue specificity. miRNAs are present in fluids either transported by RNA-binding proteins and lipoproteins or packaged into microvesicles and/or exosomes, which protect them from the degradation by ribonucleases^29^. This provides their stability at room temperature and preservation during multiple freeze-thaw cycles^30,31^. In addition, the urine collection is easy and inexpensive to acquire. These qualities provide urinary miRNAs a unique opportunity as a potential and noninvasive biomarker.

Previous studies indicated that miRNAs are important mediators of renal fibrosis and might be potential biomarkers and therapeutic targets for chronic kidney disease (CKD)^32,33^. miR-21 is one of the most extensively studied miRNAs. Previous reports have shown that miR-21 plays an important role in renal diseases, including acute kidney injury, CKD, diabetic nephropathy, renal cell carcinoma, and renal fibrosis^34^, and it is consistently up-regulated in renal diseases^35–40^. miR-21 overexpression enhances the Tgf-β1-induced epithelial-to-mesenchymal transition and aggravates renal injury in diabetic nephropathy^41^. The established role of miR-21 in tissue fibrosis leads us to believe that miR-21 may be a reliable and valuable non-invasive biomarker of hypertensive renal injury and fibrosis^35,39,40,42^. Consistent with our results, Zhou and his colleagues and other groups also suggested that miR-21 plays a key pathogenic role in kidney fibrosis, and that increases in sera and urine and associations with kidney fibrosis in a mouse model of unilateral ureteral obstruction and renal transplanted recipients^42,43^.

One study has shown that elevated levels of miR-21 and miR-142 in urine sediment of kidney transplant recipients with interstitial fibrosis and tubular atrophy were observed compared to healthy controls^24^. However, another study showed the levels of miR-21 were lower in the urine sediment of patients with IgA nephropathy compared to those in the control group, but the difference did not reach statistical significance, and urinary miR-21 levels did not correlate with urinary mRNA levels of Tgf-β1^22^. These differences may be due to different pathological processes, suggesting that we cannot generally conclude it increase or decrease in certain specific renal disease.

In the present study, specific increased miRNAs (miR-21, on day 4 of DOCA-salt treatment) from cell-free urine can be detected earlier than protein in urine (on day 8 of the DOCA-salt treatment). miR-21 levels from the area of tubulointerstitial injury and fibrosis were significantly higher than those from the tubular control (Fig. 7), which indicates that urinary miR-21 may mainly generate from tubulointerstitial injury and fibrosis. These results suggest that urinary miR-21 may be more competent than urinary protein as a non-invasive biomarker of hypertensive renal injury and fibrosis.

Currently, urinary exosomes as a biomarker have attracted the attention of researchers. Mohan and his colleagues indicated that urinary exosomal miRNA-451-5p is a potential early biomarker of diabetic nephropathy in rats^44^. Urinary exosomal miR-29 and miR-200 were significantly reduced in patients with CKD compared with controls, and, notably, the reduction correlated with the decline of renal function and the degree of tubular-interstitial fibrosis^45^. However, it is difficult to handle large lipid volumes for the isolation of exosomes. Furthermore, to date, there is still not a stable and reliable technology to isolate exosomes from urine, and the isolation of exosomes requires expensive apparatus or kits. These factors limit urinary exosomes as a utility biomarker and require further improvement. Furthermore, urine sediment mainly contains inflammatory cells and accumulated necrotic tissues or cells, which may not accurately reflect the process of disease generation and development.

In conclusion, we performed global miRNA profiling in kidneys and confirmed that urinary miR-21 may be a unique, sensitive, specific, reliable, noninvasive biomarker for early detection of hypertensive renal injury and fibrosis. A deeper understanding of this noninvasive miRNA biomarker may improve our capacity for disease monitoring and diagnosis or possibly lead to its use as therapeutic targets in clinical trials for patients with hypertensive renal injury and fibrosis.

## Methods

### Animals

Male C57bl6/J mice (20–25 g) were purchased from the experimental Animal Center of Beijing University of Medical Science (Beijing, China) and allowed to acclimate for 2 weeks. The mice were housed in a temperature-controlled room on a 12-h light/12-h dark cycle and fed standard rodent chow and tap water ad libitum. The experimental protocols were approved by the Wenzhou Medical University Committee for Laboratory Animals, and all animal treatment was consistent with the National Institutes of Health Guide for the Care and Use of Laboratory Animals. All surgeries were performed under sodium pentobarbital anesthesia and all efforts were made to minimize suffering.

### DOCA-salt hypertension model

We utilized a previously described DOCA-salt mouse model^46,47^ with minor modifications. A flank incision was made to expose the left kidney, which was ligated and removed. The incision was then sutured. After one week of recovery, a 21-day-release DOCA pellet containing 75 mg of DOCA (Innovative Research of America, Sarasota, FL) was implanted subcutaneously by incision of the right flank under light ether anesthesia. The mice were then allowed to recover in a warm cage. Control animals were sham operated. All of the animals (DOCA and control groups) were fed an 8% NaCl diet starting at the third day before DOCA treatment.

The mice were placed into metabolic cages, and urine was collected for 24 hours for the measurement of creatinine, albumin, and miRNAs before and after DOCA pellets were implanted for 2 days, 4 days, and 8 days. Urine samples collected for the miRNA assay were mixed with an equal volume of the denaturing solution in the *mir*Vana PARIS Kit (Ambion, TX) and maintained at −80 °C until purification. The mice were then sacrificed by dissecting the abdominal artery and bleeding under deep anesthesia. After measuring the body and kidney weight, the organs were decapsulated. One fraction of the kidney was put in 4% formaldehyde for fixation, whereas the other fraction of kidney was left for total RNA purification.

### Urinary analyses

The urine albumin, NAG, creatinine and serum creatinine concentrations were measured with an ELISA kit (Exocell; Shibayagi, Gunma, Japan), a NAG test (Nanjing Jiancheng Bioengineering Institute; Nanjing, China) and Quantichrom^TM^ creatinine assay kit (Bioassay system; CA), respectively. For all of the assays, the samples were run in duplicate and the results were averaged.

### Renal histology and immunohistochemical analyses

The tubulointerstitial lesions and fibrosis were evaluated in renal tissue sections (5 μm) with Masson trichrome staining. Ten fields from the cortical areas were selected randomly per mouse. Tubulointerstitial lesions were defined as the accumulation of tubular dilatation/atrophy, tubule vacuole and cast formation, interstitial edema, and epithelial cell necrosis^47^. Tubulointerstitial fibrosis was defined as the accumulation of the extracellular matrix (stained blue). The extent of tubulointerstitial injury and fibrosis was defined as a ratio relative to the entire cortical area with ImageJ software (National Institutes of Health, Bethesda, MD).

Immunohistochemical staining was performed as described previously^47^. Deparaffinized kidney sections were heated for 20 min at 121 °C in a 10-mM citric acid solution for antigen retrieval and then incubated with antibody against F4/80 (Santa Cruz Biotechnology, Santa Cruz, CA). The primary antibody was detected using the Histofine Simple Stain MAX-PO (mouse) kit (Nichirei, Tokyo, Japan) and peroxidase stain DAB kit (Nacalai Tesque, Kyoto, Japan). The nuclei were stained with hematoxylin.

### Laser capture microdissection (LCM)

The laser capture was performed with the Leica LMD 6000 Laser Microdissection system. The kidney tissues were rapidly dissected in an RNAse-free environment, immediately embedded in O.C.T. compound (Sakura Finetek), sectioned at 10 μm and mounted on PEN-membrane slides (nuclease and human nucleic acid free) (Lecia-REF11505189). To visualize the areas of interest, tissue sections were stained with hematoxylin and eosin. To allow for proper excision performance, the slides were completely air dried before microdissection. Regions of interest from the specimen were captured in 0.5-mL microcentrifuge tubes (Eppendorf Safe-Lock Tubes). Approximately 200,000–400,000 μm^2^ of each tissue type was collected from each sample.

### RNA purification

The total RNA was extracted from the kidney with TRIzol regent (Invitrogen) as previously described^47^. Total urine RNA and RNA from the LCM samples were isolated with the *mir*Vana PARIS Kit (Ambion, TX) according to the manufacturer’s protocol. Briefly, total urine RNA was purified from 100 µL of urine and ultimately eluted into 100 µL of RNase-free water. The RNA was then precipitated with ethanol in the presence of a polyacrylamide polymer solution (Ethachinmate; Nippon Gene) and resuspended in 20 µL of RNase-free water.

### MicroRNA array analysis

To assess the different signature of the miRNAs of the mouse kidney induced by DOCA-salt treatment and the control, we profiled the production of 588 miRNAs in the kidney with the ABI TaqMan MicroRNA Array kit (Applied Biosystems) according to the manufacturer’s protocol. In brief, 333 ng of mouse kidney total RNA was reverse-transcribed with megaplex RT primers (Megaplex RT Rodent Pool A or B) followed by a pre-amplification reaction with megaplex preamp primers; subsequently, a real-time PCR with TaqMan Rodent MicroRNA Array (Pool A or B) was performed on an Applied Biosystems 7900HT System. SDS software V2.3, and RQ Manage1.2 (Applied Biosystems) were used to obtain the comparative threshold cycle (Ct) value. U6 small nuclear RNA included in the TaqMan Rodent MicroRNA Array was used as an endogenous control.

### Quantification of miRNAs by real-time reverse-transcription PCR (RT-PCR)

Stem-loop real-time PCR was used to quantify the expression of miRNAs (miR-21, miR146b, miR-132 and miR-155) with a TaqMan MicroRNA real-time RT-PCR kit according to the manufacturer’s protocol. The reverse-transcription reaction was initiated with 1 μg of kidney-derived total RNA or with 10 μL of urine-derived total RNA (from 100 μL of urine). Negative controls were included with every real-time RT-PCR assay. The 7500 fast real-time PCR system (Applied Biosystems) was used for amplification and detection. The 5 S rRNA was used as an internal control.

### Statistical analysis

The data were analyzed using a one-way analysis of variance (ANOVA) and then Tukey’s HSD test using the JMP statistical analysis package (SAS Institute, Cary, NC). *P* < 0.05 was considered significant. The data are presented as the means ± SD. Microarray analysis involved multiple sample analyses, including normalization, data adjustment, and clustering. Hierarchical clustering of the experimental variation in the gene expression was determined using software programs developed at Stanford University^48^. The cluster algorithm was set to complete the linkage clustering using the uncentered Pearson correlation.
